# CT predicts intraprocedural hemodynamics with computational fluid dynamics in TMVR-ineligible patients undergoing M-TEER

**DOI:** 10.3389/fcvm.2025.1665934

**Published:** 2025-11-19

**Authors:** Johannes Kirchner, Muhammed Gerçek, Hazem Omran, Mohammad Kassar, Christoph Gräni, Fabien Praz, Felix Rudolph, Volker Rudolph, Tanja K. Rudolph

**Affiliations:** 1 Clinic for General and Interventional Cardiology/Angiology, Herz-und Diabeteszentrum NRW, Ruhr-Universität Bochum, Bad Oeynhausen, Germany; 2 Department of Cardiology, Inselspital Bern, Bern University Hospital, Bern, Switzerland

**Keywords:** mitral regurgitation, transcatheter edge-to-edge repair, computational fluid dynamics, hemodynamic, CT

## Abstract

**Background:**

Hemodynamic outcomes in patients undergoing transcatheter mitral edge-to-edge repair (M-TEER) are difficult to predict. Computational fluid dynamics (CFD) is frequently used in biomedical engineering to simulate blood flow patterns under various conditions.

**Objectives:**

We developed a standardized workflow for individualized CFD analyses to predict postinterventional mitral valve gradients and residual regurgitation following TEER.

**Methods:**

Twenty patients with severe mitral regurgitation (MR) from two high-volume centers underwent full-cycle cardiac computed tomography before intervention. Based on the specific valve morphology, individualized CFD simulations were performed to calculate MR volumes prior to intervention and estimate hemodynamics after M-TEER.

**Results:**

CFD analyses (mean age 80 ± 4 years, 55% male) showed excellent correlation between baseline proximal isovelocity surface area (PISA)-based MR volumes, median 40 ml [interquartile range (IQR): 30–49 ml], and CFD-based calculation, median 30 ml (IQR: 27–54 ml; *R* = 0.917; *P* < 0.001), as well as between baseline effective regurgitant orifice area (EROA) assessed in transesophageal echocardiography (TEE) and CFD-measured EROA (*R* = 0.869; *P* < 0.001). After device implantation, the correlation between intraprocedural TEE-measured and CFD-estimated residual MR (*R* = 0.949; *P* < 0.001) and EROA (*R* = 0.841; *P* < 0.001) remained robust. Median postinterventional diastolic pressure gradient (TEE) was 2.8 mmHg (IQR: 1.7–4.0), which closely correlated with the CFD-estimated gradient of 1.4 mmHg (IQR: 2.3–4.5, *R* = 0.905; *P* < 0.001).

**Conclusions:**

This is the first study to use a standardized CFD workflow for MR evaluation in patients undergoing TEER. In the future, CFD-based analyses may serve as a key diagnostic tool for procedural planning of TEER.

## Introduction

The establishment of catheter-based techniques for mitral valve repair offers treatment options for a large number of patients who would otherwise be denied invasive treatment or are at high risk for conventional surgery. In patients with severe mitral regurgitation (MR), transcatheter mitral valve replacement (TMVR) is a viable treatment option. In the evaluation process of TMVR, computed tomography (CT) is mandatory to test for TMVR eligibility. Unfortunately, up to 90% of screened patients are reported to be ineligible due to anatomic reasons, prior aortic valve therapy, or excessive frailty (1). Untreated patients carry a high risk for hospitalization and death (1), and edge-to-edge repair (TEER) of the mitral valve is often used as a bailout therapy in these patients. Despite the growing body of experience in TEER, achieving minimal residual MR can be challenging due to complications such as iatrogenic mitral stenosis. Residual MR is associated with a higher risk for death or heart failure hospitalizations after TEER (2,3).

Computational fluid dynamic (CFD) is frequently used in biomedical research to study blood flow patterns under various conditions. These models provide detailed 3D insights into local blood flow phenomena in patients with heart failure (4). We sought to predict procedural outcomes using CFD simulations in patients who had received CT for TMVR but were deemed ineligible for TMVR.

## Methods

### Study design

Twenty patients from two high-volume centers were included in this study. All patients were deemed unsuitable for conventional heart surgery due to high surgical risk. For evaluation of mitral valve replacement, the patients underwent full-cycle cardiac CT. The study was approved and overseen by the local ethics committee, and informed consent for retrospective study inclusion was waived.

### Echocardiographic assessment and procedure

All patients received transthoracic echocardiograms (TTE) within 24–48 h prior to the intervention, with assessments performed according to current American Society of Echocardiography (ASE) recommendations. The severity of MR was graded according to the 4-grade scheme proposed by the ASE. The TEER procedure was performed under general anesthesia with interventional guidance by transesophageal echocardiography (TEE) and fluoroscopy via femoral vein access. Technical success was defined as successful device delivery, deployment, and positioning of the device, absence of procedural mortality, and freedom from emergency surgery related to the device.

### Valve mapping

**Figure 1:**
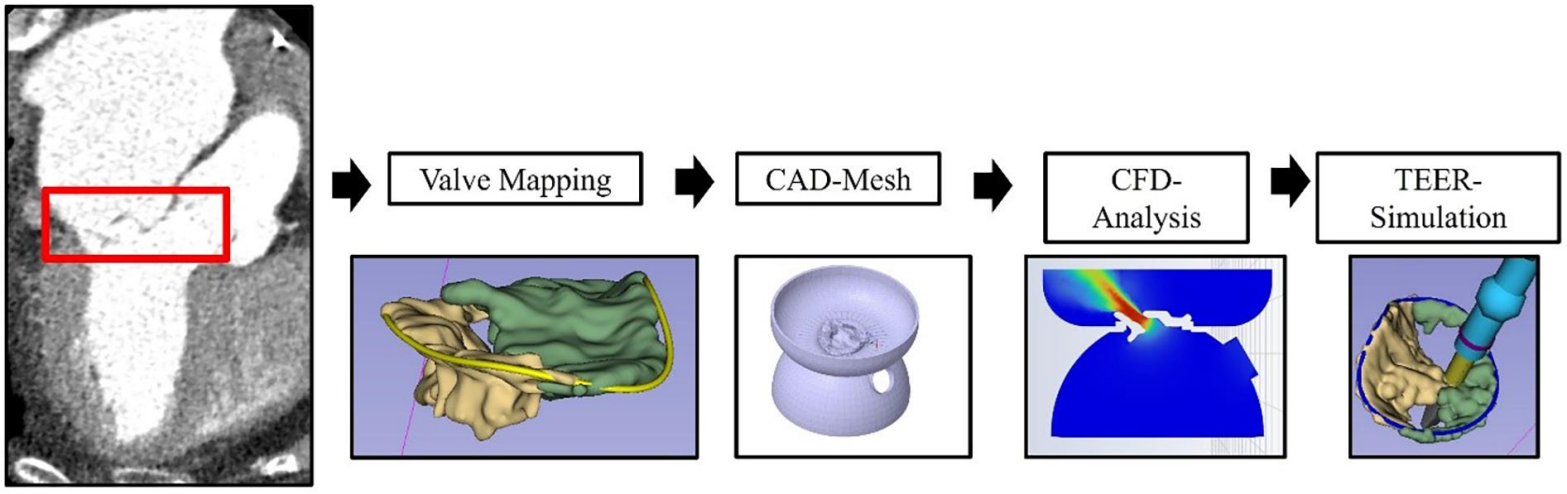
Workflow for CFD analysis. Illustration of the workflow for CFD analysis based on 3D computed tomography imaging. CAD, computed-aided design; CFD, computational fluid dynamics; TEER, transcatheter edge-to-edge repair.

The workflow for the CFD-based valve assessment is shown in Figure 1. Multi-detector CT 3D modeling image acquisition was performed using an electrocardiography-gated phase-specific protocol with iodinated contrast medium. CT image reconstruction was performed for the entire cardiac cycle at 5%–10% increments of the R–R interval, depending on the local protocol. The reconstructed slice thickness was <1 mm.

CT scans were imported to 3D Slicer 5.6.1, a free and open-access software for image processing (https://www.slicer.org/) (5). The shape of the mitral valve was manually marked in mid-systole and mid-diastole. To estimate the impact of the device on the valve morphology, the Slicer device tool was used to approximate changes in leaflet morphology with respect to the size of the device.

The 3D models were then exported to Ansys SpaceClaim (Ansys Inc., USA) as an STL files. The valve model was implemented in a standardized model covering the mitral valve annulus and left ventricular outflow (LVOT). The size of the LVOT was derived from the CT scan in the mid-systolic phase. Then this 3D-model was “shrinkwrapped” using 0.5 mm net size and exported to Ansys Fluent 19.0 (Ansys Inc., USA).

The CT-based left ventricular stroke volume was derived from heart.ai., which is a cloud-based platform for artificial intelligence-based image analysis. Mean diastolic and systolic flow rates were calculated by dividing the left ventricular stroke volume by the duration of the diastole or systole, respectively.

### CFD simulation

Volumetric meshes were built in Ansys Fluent. The model was prepared for flow simulations using a watertight workflow. The computed-aided design (CAD) mesh was composed of tetrahedral elements in the core and three inflation layers with prism-shaped cells along the wall. The mesh had minimum edge lengths of 0.1 mm.

Mean diastolic and systolic flow rates were used as the inlet velocities in diastole and systole, respectively. Taking into account the individual diastolic and systolic valve morphology, gradients and velocity were calculated.

Blood flow through the regurgitant mitral valves was simulated at a single time point during mid-systole. Blood was modeled as an incompressible Newtonian fluid with a density of *ρ* = 1,060 kg·m^−3^ and a dynamic viscosity of *η* = 0.004 Pascal·second (6).

Solutions were solved numerically with Ansys Fluent 19.0 under steady-state conditions with laminar inlet flow. In diastole, only one inlet orifice and one outlet orifice with no specific pressure boundaries were defined. In systole, the same pressure of the inlet and outlet was set in every simulation [given left atrial pressure 10 mmHg, left ventricle 120 mmHg, LVOT 80 mmHg]. One inlet orifice (left ventricle) and two outlet orifices (left atrium and LVOT) were defined. The CFD-based results were compared with the intraprocedural TEE-based results during TEER. Calculated regurgitant volumes and mean pressure gradients (MPG) using CFD were compared with results using the proximal isovelocity surface area (PISA) method and velocity acceleration via the mitral valve orifice at the beginning of the procedure and after the first device implantation.

### Statistical analysis

Statistical analysis was performed using SPSS Statistics 27 (IBM Corp.). Continuous variables were reported as mean ± standard deviation if normally distributed and as median and interquartile range (IQR) if not normally distributed. Categorial variables were presented as frequencies and percentages. Bland–Altman analysis was performed for comparison of AI-based and conventional CT analysis by calculating mean difference, standard deviation of difference (SDD), and limits of agreement (LoA). Pearson correlation coefficients were calculated. A *p*-value of <0.05 was considered statistically significant.

## Results

### Study population

Baseline clinical characteristics of the enrolled patients are shown in Table 1. The mean age was 82 ± 4 years, and 55% were male. Most patients were severely symptomatic, with 90% presenting in New York Heart Association class ≥ III. The median EuroScore II was 6.8% (IQR: 4.8–11.5). The cause of MR was primary in 50% of cases and secondary in 50% of cases. In baseline TTE, the mean left ventricular ejection fraction was 49% ± 11%, and the MR grade was 2+ in 5%, 3+ in 40%, and 4+ in 55% of the patients (Table 2).

**Table 1:** Characteristics of the patients at baseline.

Baseline data	All
Age (years), mean ± SD	82 ± 4
Male, *n* (%)	11 (55)
Body mass index (kg/m^2^), mean ± SD	28 ± 6
EuroScore II (%)	6.8 (4.8–11.5)
GFR (ml/min), mean ± SD	45 ± 23
NTpro-BNP (pg/ml), median (IQR)	2,260 (1,750–4,560)
Coronary artery disease, *n* (%)	12 (60)
Diabetes mellitus, *n* (%)	4 (20)
CABG, *n* (%)	3 (15)
NYHA, *n* (%)	
I	
II	2 (10)
III	15 (75)
IV	3 (15)
Arterial hypertension, *n* (%)	18 (90)
COPD, *n* (%)	1 (5)
Atrial fibrillation, *n* (%)	19 (95)

CABG, coronary artery bypass graft; COPD, chronic obstructive pulmonary disease; EROA, effective regurgitation orifice area; GFR, glomerular filtration rate; NTpro-BNP, N-terminal prohormone of brain natriuretic peptide; NYHA, New York Heart Association functional classification.

**Table 2:** Baseline echocardiographic data and procedural outcome data.

Baseline echocardiographic data	All
LVEF (%), mean ± SD	49 ± 11
MR baseline, *n* (%)	
2+	1 (5)
3+	8 (40)
4+	11 (55)
MR etiology, *n* (%)	
Primary	10 (50)
Secondary	10 (50)
TR baseline, *n* (%)	
1	9 (45)
2	5 (25)
3	5 (25)
4	0 (0)
5	1 (5)
Procedural outcome data	
Number of implanted devices, *n* (%)	
0/failed implantation	2^a^ (10)
1	14 (67)
2	3 (14)
3	2 (10)
MR at the end of procedure, *n* (%)	
0/trace	6 (30)
1+	9 (45)
2+	2 (10)
3+	2 (10)
4+	1 (5)
Type of devices, *n* (%)	
Pascal	3 (14)
Pascal Ace	6 (29)
XTw	6 (29)
XTr	2 (10)
XT	1 (5)
NT	1 (5)
NTr	2 (10)
EROA post procedure (mm^2^), median (IQR)	10 (2–13)
Regurgitation volume post procedure (ml), median (IQR)	12 (8–17)
Computed tomography data, mean ± SD	
Mitral annulus area systole	1,595 ± 379
Mitral annulus area diastole	1,630 ± 380
Mitral annulus perimeter systole	141 ± 16
Mitral annulus perimeter diastole	144 ± 15

EROA, effective regurgitation orifice area; LVEF, left ventricular ejection fraction; MR, mitral regurgitation; TR, tricuspid regurgitation.

a In one case, device implantation resulted in relevant mitral stenosis, and in one case, residual MR was too prominent for permanent device implantation.

The technical success rate was 90%. In two patients, device implantation failed—one due to relevant mitral stenosis and the other due to insufficient MR reduction. Comparison of the baseline and postprocedural TEE demonstrated a significant reduction of MR grade, as well as effective regurgitation orifice area (EROA) and regurgitation volume (RV) (*P* < 0.001; Central Illustration).

**Central Illustration:**
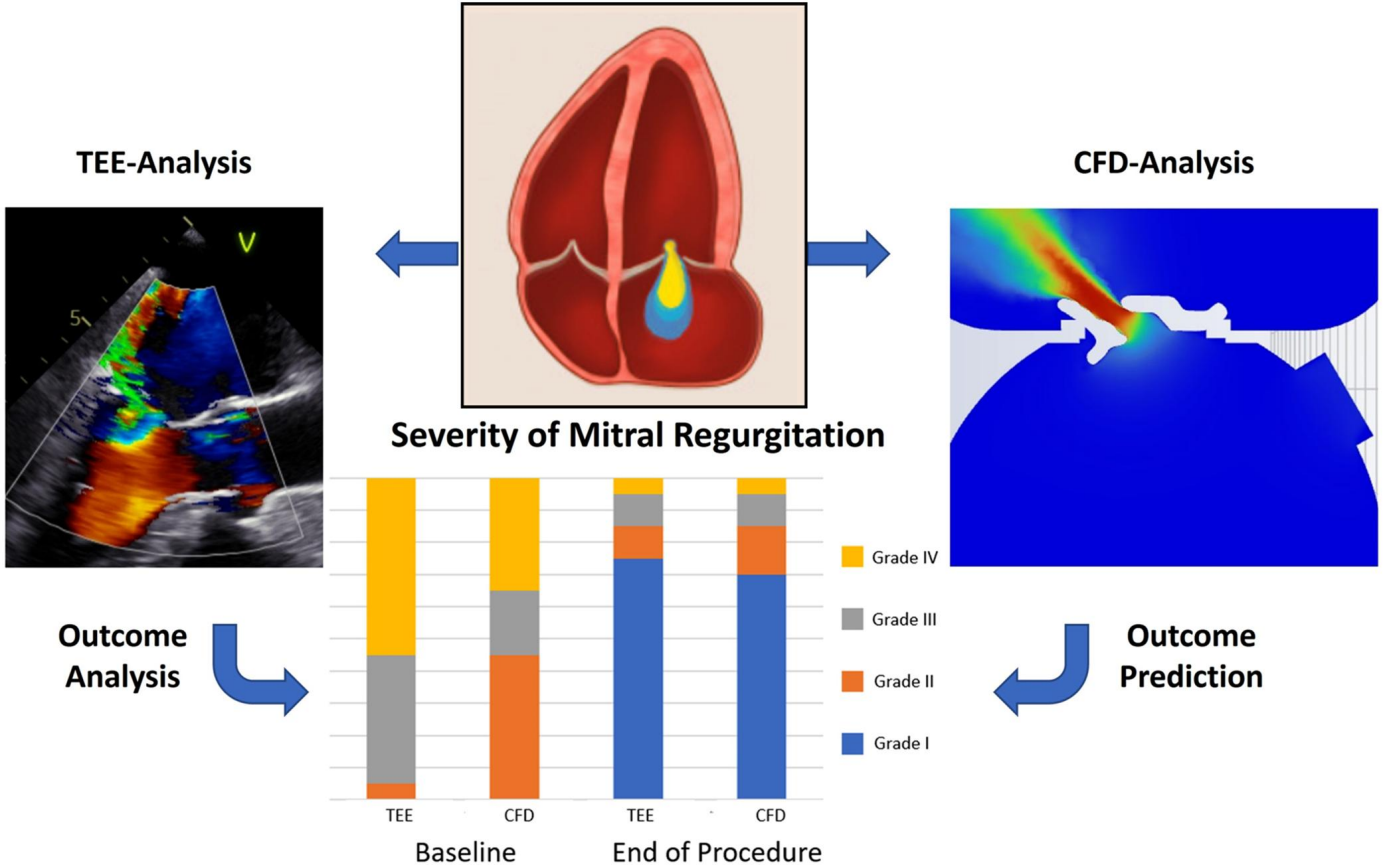
Prediction of hemodynamics with computational fluid dynamics in mitral valve edge-to-edge repair. Computational fluid dynamics was used to calculate the severity of mitral regurgitation, residual mitral regurgitation, and mean diastolic gradient after device implantation. Baseline and postprocedural results of the CFD calculation showed a strong correlation to TEE-measured results at baseline and at the end of the procedure. TEE, transesophageal echocardiography; CFD, computational fluid dynamics.

### Baseline and postprocedural hemodynamic

Correlations and differences of baseline and postprocedural measurements from both methods are shown in Supplementary Table S1 and Figure S1. An exemplary illustration showing a TEE- and CFD-visualized regurgitation jet is presented in the Central Illustration. Baseline median MR volumes were 40 (IQR: 30–49) ml measured in TEE, and median CFD-based calculation was 30 (IQR: 27–54) ml which was closely correlated with each other (*R* = 0.917; *P* < 0.001). Consequently, TEE-measured baseline EROA [23 (IQR: 20–35) mm^2^] and CFD-measured EROA [19 (IQR: 13–27) mm^2^] also revealed a good correlation (*R* = 0.869; *P* < 0.001). After device implantation, the correlation between intraprocedural TEE-measured and CFD-estimated correlation for residual MR (*R* = 0.949; *P* < 0.001) and EROA (*R* = 0.841; *P* < 0.001) remained robust. With a mean difference of 0.6 ± 1.3 mmHg, postinterventional TEE-measured diastolic pressure gradient was in close correlation with the CFD-estimated gradient (*R* = 0.905; *P* < 0.001). In the two patients with failed device implantation due to relevant mitral stenosis, the MPG were 13 and 8 mmHg, respectively, and previously calculated values using CFD were 8 and 5 mmHg.

## Discussion

This study is pioneering in its use of 3D CT imaging combined with CFD simulations to evaluate MR, providing a quantitative assessment that can complement traditional echocardiographic evaluations. The ability to visualize valve morphology and assess hemodynamic impacts before procedures represents a significant advancement in pre-procedural planning.

### Quantification of baseline mitral regurgitation

This is the first study that uses 3D CT-based image analysis for CFD simulations in MR patients and compares this method to PISA-measured regurgitant volumes. It is notable that for the classification of MR, more echocardiographic factors than RV or EROA are taken into account. Similar to echocardiography, structural effects of MR on the left heart, such as atrial and ventricular dilation, can be assessed in CT (7, 8). Qualitative assessment of the mitral valves with large flail leaflets or restrictive leaflets might also be visualized in CT but small coaptation deficits are still challenging to detect.

Our approach uses the specific valve morphology and CFD calculation to estimate regurgitation volumes. As with echocardiography, this new quantitative method can lead to an over- or underestimation of MR. Most patients with severe MR suffer from atrial fibrillation (95% in our study) which impacts the calculation of RV due to ever-changing left ventricular stroke volumes. An advantage of CFD-based MR evaluation is the high temporal and spatial resolution of CT scans compared with TEE-based 3D valve reconstruction methods (9). This enables the calculation of MR volumes with respect to the individual valve morphology, whereas echocardiographic calculation mainly relies on (color-flow) Doppler measurements. Given the different nature of the two methods, the relatively small differences in our findings regarding MPG and RV underline the accuracy and validity of this new method.

### Mean pressure gradient and residual regurgitation volumes after TEER

The clinical success of a procedure heavily depends on its hemodynamic effects. In our study, technical success was achieved in 90% and 85% of the patients had residual MR ≤ 2+ at the end of the procedure.

Makkar et al. have reported that a mean pressure gradient of <5 mmHg and minimal residual MR are most favorable in primary mitral regurgitation (PMR) after TEER, whereas a gradient of >5 mmHg and moderate residual MR or unsuccessful procedures were associated with a significantly increased 1-year mortality. Whether these MPG cutoff values can be attributed to secondary mitral regurgitation (SMR) is under debate (10, 11). However, before TEER procedures, it is often unclear and difficult to predict whether and to what extent the devices will lead to a significant increase in gradients or a satisfactory reduction of MR volume (7). Using the baseline CT images, we simulated TEER device implantations. For these CFD calculations, device implantation was simulated with a reduction of the coaptation deficit only in close proximity to the device. For diastolic gradients, postinterventional gradients correlated well between CFD and echo-based MPG with the difference between both procedures being <2 mmHg in 90% of the cases. Therefore, accurate prediction of diastolic MPG after TEER might be possible in larger cohorts.

Furthermore, we investigated whether the reduction of regurgitant volumes can be simulated with CFD. Using virtual device implantation, coaptation gaps were only closed in the assumed grasping area without further impact on valve morphology. This also led to excellent correlations between TEE-measured CFD-estimated residual MR. Hence, this approach has the potential to estimate residual MR prior to the procedure and therefore refashion pre-procedural planning.

### Perspective for CFD simulation in valvular heart disease

Therapy planning tools for transcatheter interventions are tremendously needed when it comes to individualized device choice, positioning, and outcome prediction (7). Other than CT-based prosthesis selection in transcatheter aortic valve replacement (TAVR), there is no standardized workflow for device selection in MR procedures. Ideally, pre-procedural imaging enables procedure planning and assesses the risk of iatrogenic mitral stenosis, residual MR, or outflow tract obstruction. This may be approached with CFD analyses with respect to hemodynamic parameters. Ultimately, physicians may better understand a patient's unique anatomy and can bench test several catheter-based interventions. In addition, this approach could be integrated into TEER procedures when residual MR is present after placement of the first device. It may help assess whether an additional device should be implanted, where it should be positioned, and what the hemodynamic consequences might be.

## Limitations

Despite the promising results, the study is limited by its small cohort size and the need for further validation in larger populations. The complexity of preparing CFD simulations and the reliance on multiple software tools were noted as challenges. For the interpretation of diastolic gradients, it is important to recall that the simplified Bernoulli equation is MPG = 4v^2^. This means that even minimal increases in mean flow velocities—from 1.1 to 2.0 m/s—can result in a substantial rise in diastolic gradients, ranging from approximately 4.8 to 12 mmHg. Consequently, in both TEE and CFD-based measurements, relatively small changes in flow velocity may significantly impact the final gradient, which partially determines a patient's suitability for TEER. Furthermore, several studies have reported an annuloplasty-like effect on the mitral valve annulus caused by TEER. It is unclear whether this “annuloplasty-effect” translates into a relevant reduction of RV and was not taken into account in our analysis.

However, the potential for integrating artificial intelligence to automate certain aspects of this workflow could enhance its feasibility in clinical practice.

## Conclusion

This study establishes a CT-based and CFD-standardized workflow for predicting regurgitant volumes and hemodynamic changes in patients ineligible for TMVR undergoing TEER. By leveraging CFD analyses, clinicians may gain a better understanding of individual patient anatomy and enhance procedural planning, ultimately aiming to reduce residual MR and improve outcomes in patients with mitral valve disease. Further research with larger cohorts will be necessary to validate these findings and optimize the workflow for clinical use.

## Data Availability

The original contributions presented in the study are included in the article/Supplementary Material, further inquiries can be directed to the corresponding author.
