# Supplementary material for: CT predicts intraprocedural hemodynamics with computational fluid dynamics in TMVR-ineligible patients undergoing M-TEER
Source: Front Cardiovasc Med. 2025 Nov 19;12:1665934. doi: 10.3389/fcvm.2025.1665934 (PMC12672551; doi:10.3389/fcvm.2025.1665934)
Supplement: Supplementary file 1 [file Table1.pdf]

Supplementary Table 1. Comparison between TEE-measured and CFD-calculated mitral regurgitation and mean gradient.

	Echo-measured	CFD predicted	Pearson correlation Index	Mean / Median difference	P-Value for Pearson Correlation
Mean pressure gradient, mmHg, median (IQR)	1.4 (0.9; 2)	0.9 (0.6; 1.3)	0.059	0.4 (0.1; 0.9)	0.803
Mean pressure gradient after device implantation, mmHg, median (IQR)	3.6±2.7	3.9±1.8	0.905	0.6±1.3	<0.001
Regurgitation Volume, ml, median (IQR)	40 (30; 49)	30 (27; 54)	0.917	0 (-4; 2)	<0.001
Regurgitation Volume after device implantation, ml, median (IQR)	13 (8; 23)	11 (3; 21)	0.949	2±6	<0.001
EROA, mm², median (IQR)	23 (20; 35)	19 (13; 27)	0.869	3±9	<0.001
EROA after device implantation, mm², median (IQR)	10 (4; 10)	5 (2; 11)	0.841	3±5	<0.001

Values are mean ± SD or median (IQR).

EROA, effective regurgitation orifice area
