# Supplementary material for: CT predicts intraprocedural hemodynamics with computational fluid dynamics in TMVR-ineligible patients undergoing M-TEER
Source: Front Cardiovasc Med. 2025 Nov 19;12:1665934. doi: 10.3389/fcvm.2025.1665934 (PMC12672551; doi:10.3389/fcvm.2025.1665934)
Supplement: Supplementary file 2 [file Image1.pdf]

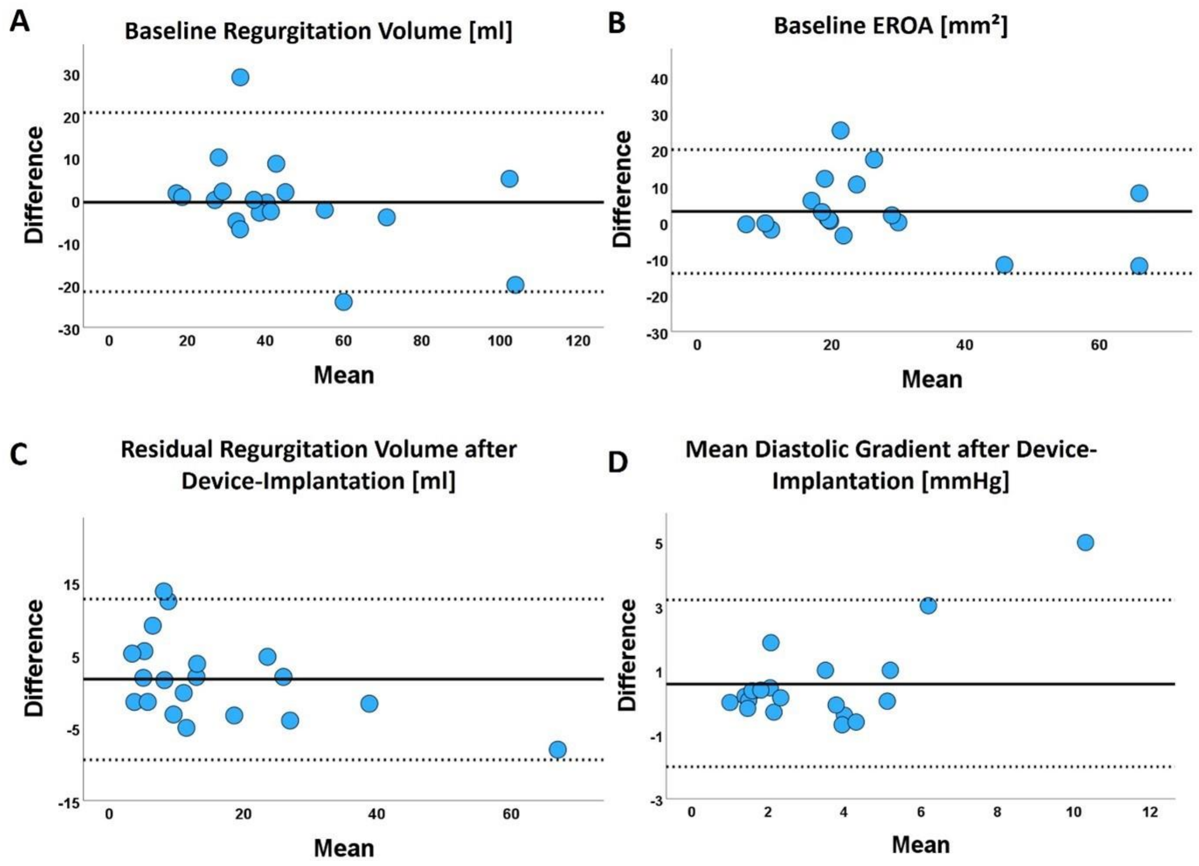

Supplementary Figure 1. Bland-Altman-Blots comparing TEE- and CFD-measurements.

Bland-Altman-Blots comparing TEE-measured and CFD-calculated baseline MR volumes (A) and calculated baseline EROA (B) as well as residual MR volumes (C) and postinterventional mean diastolic gradient (D)
